# Selective eradication of human non-small cell lung cancer cells using aptamer-decorated nanoparticles harboring a cytotoxic drug cargo

**DOI:** 10.1038/s41419-019-1870-0

**Published:** 2019-09-20

**Authors:** Shira Engelberg, Einat Netzer, Yehuda G. Assaraf, Yoav D. Livney

**Affiliations:** 1 The Laboratory of Biopolymers for Food and Health, Department of Biotechnology and Food Engineering, Technion – Israel Institute of Technology, 3200000 Haifa, Israel; 2 The Fred Wyszkowski Cancer Research Laboratory, Department of Biology, Technion – Israel Institute of Technology, 3200000 Haifa, Israel

**Keywords:** Targeted therapies, Drug development

## Abstract

Targeted cancer therapy is currently the leading modality to enhance treatment selectivity and efficacy, as well as to minimize untoward toxicity to healthy tissues. Herein, we devised and studied nanoparticles (NPs) composed of the biocompatible block-copolymer PEG-PCL entrapping the hydrophobic chemotherapeutic drug paclitaxel (PTX), which are targeted to human non-small cell lung cancer (NSCLC) cells. To achieve selective NSCLC targeting, these NPs were decorated with single-stranded oligonucleotide-based S15 aptamers (S15-APTs), which we have recently shown to serve as efficient tumor cell targeting ligands. Prepared without using surfactants, these 15 nm PEG-PCL/PTX NPs entered NSCLC cells via clathrin-mediated endocytosis. These NPs demonstrated efficient encapsulation of PTX, high selectivity to- and potent eradication of human A549 NSCLC cells, with a remarkable half maximal inhibitory concentration (IC_50_) of 0.03 μM PTX. In contrast, very high IC_50_ values of 1.7, 4.2, 43, 87, and 980 µM PTX were obtained towards normal human bronchial epithelial BEAS2B, cervical carcinoma HeLa, colon adenocarcinoma CaCo-2, neonatal foreskin fibroblast FSE, and human embryonic kidney HEK-293 cells, respectively. These results demonstrate 2–5 orders of magnitude difference in the selective cytotoxicity towards NSCLCs, reflecting a potentially outstanding therapeutic window. Moreover, the dual utility of aptamer-decorated NPs for both drug stabilization and selective tumor targeting was studied by increasing APT concentrations during NP “decoration”. The optimal aptamer density on the surface of NPs for selective targeting, for high fluorescence diagnostic signal and for maintaining small particle size to enable endocytosis, was achieved by using 30 nM APTs during NP decoration. Collectively, our findings suggest that these APT-decorated NPs hold great preclinical promise in selective targeting and eradication of human NSCLC cells without harming normal tissues.

## Introduction

Lung cancer is the primary cause of cancer-related mortality in the United States (U.S)^1^. It accounted for 26% of all cancer-related deaths in the U.S in 2017^2^. Non-small cell lung cancer (NSCLC) is the predominant class (~85%) of lung cancers^3^. Chemotherapy is currently the primary treatment modality in NSCLC^4^. The major deleterious impact of nonselective chemotherapy is the untoward side effects to healthy tissues including neutropenia. Due to low levels of neutrophils, neutropenic cancer patients are exposed to pathogen infections occurring in ~half of the cancer patients receiving nonselective-chemotherapy, resulting in 10% mortality^5^.

Major efforts have been recently focused on the development of targeted cancer therapeutics to circumvent the toxic side effects of chemotherapy and to enhance anti-tumor activity. The main targeting strategies include passive targeting, based on the enhanced permeation and retention (EPR) effect^6^, and active targeting, based on molecular recognition using targeting ligands, which bind to proteins predominantly (over)expressed on the surface of cancer cells. These targeting molecules include e.g. folic acid, antibodies, or aptamers (APTs), also known as chemical antibodies^7,8^. Decorating nanoparticles (NPs) with molecular recognition ligands is a promising strategy to obtain active targeting, allowing for highly selective treatment and minimization of untoward side-effects^9^.

APTs, made of single-stranded oligonucleotides, are novel molecular probes attracting major interest as important ligands, which can be synthesized and directed against a wide range of disorders including various human malignancies. By spontaneously folding into a distinct tertiary structure, an APT can recognize a unique target with high affinity and specificity^10^. In this respect, the S15-APT is an 85-base long single-stranded DNA, selected for specific binding to NSCLC^11^. APTs offer key advantages over conventional antibodies as elaborated previously^10–12^. APTs have been widely studied from various aspects, due to their great potential as therapeutic targeting components for diverse biomedical applications including cancer diagnosis, anti-tumor therapeutics^13,14^, biomarker identification^13,15^, and active targeting ligands for advanced drug delivery systems^16,17^. APTs may be tailored for any cancer cell type, or even for a tumor in a given cancer patient, hence they pave the way towards precision medicine.

Polymeric biodegradable NPs have attracted a great interest as effective drug delivery vehicles^18^. Amphiphilic block copolymers, which undergo self-assembly in aqueous solutions to form micelles, represent a well-established approach for the preparation of micellar drug carriers. They display high loading capacity (LC) and thermodynamic stability under physiological solutions, enabling prolonged circulation in the bloodstream for passive targeting by the EPR strategy^6^, thus enhancing the effective drug concentration within the tumor. The hydrophilic shell and small size (preferably <200 nm) minimize their uptake by the reticuloendothelial system (RES)^19,20^. PEGylation improves the pharmacokinetic and pharmacodynamic profiles of the nano-carrier, preventing its elimination, thereby increasing its circulation time^21^. Based on these considerations, the preferred size range of NPs aimed at drug targeting has been suggested to be 10–200 nm^20,22^. The ideal size, at which NPs escape renal exclusion and RES uptake, show enhanced cell permeability into solid tumors via passive diffusion, and undergo endocytosis into malignant cells is ~10–50 nm^23,24^.

A major chemoresistance mechanism is multidrug resistance (MDR)^25,26^. An effective modality to overcome MDR is internalization of actively targeted NPs via selective endocytosis, consequently evading MDR efflux pumps residing in the plasma membrane, including P-glycoprotein (P-gp/ABCB1)^27,28^ and breast cancer resistance protein (BCRP/ABCG2)^29–31^. Selectively targeted delivery via endocytosis enables reducing drug doses and untoward toxicity, while maximizing treatment efficacy.

Herein we developed a targeted delivery platform for hydrophobic chemotherapeutics (e.g. Paclitaxel (PTX)), based on polyethylene-glycol (PEG) conjugated to polycaprolactone (PCL) to form PEG-PCL micelles, decorated with APTs as the targeting moieties. These APT-NPs selectively delivered PTX to model target NSCLC A549 cells, and efficiently eradicated them, while avoiding cytotoxicity to normal human bronchial epithelial BEAS2B, neonatal foreskin fibroblast FSE and human embryonic kidney cells, and also to cervical carcinoma HeLa, colon adenocarcinoma CaCo-2. Confocal laser microscopy revealed that these 15 nm APT-NPs underwent internalization into target NSCLC cells via clathrin-mediated endocytosis, suggesting that they may readily overcome cancer MDR^23,25^.

## Results

### Self-assembly of the PEG-PCL delivery system

**Fig. 1:**
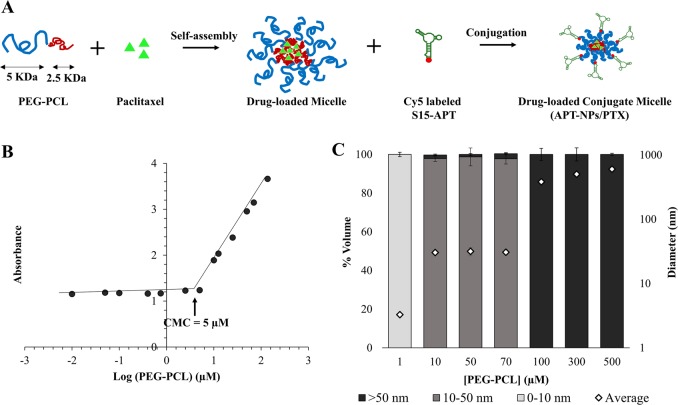
Nanoparticle Structure, Self-assembly and size distribution. **a** Schematic structure of the NPs; **b** Determination of the CMC of PEG-PCL in water; **c** Size distribution of polymer micelles. Volume-weighted percent of PEG-PCL and overall average diameter (◊). Values presented are means ± standard error (SE).

To form NPs suitable for endocytosis by target NSCLC cells, we aimed at forming NPs with a size <50 nm^23,24^. PEG-PCL block-copolymer (5 KDa:2.5 KDa) self-assembled micelles were prepared by surfactant-free nanoprecipitation (Fig. 1a)^32^. The critical micelle concentration (CMC) of the polymer was determined by following the enhancement of the absorbance of a hydrophobic dye 1,6-diphenyl-1,3,5-hexatriene (DPH) upon entrapment in micelles^56^. The 378 nm to 400 nm absorbance ratio was plotted against increasing PEG-PCL concentrations. When increasing the polymer concentration, above the CMC, the relative absorbance increased logarithmically due to dye entrapment in the hydrophobic core of the micelles; the CMC was found to be 5 μM (Fig. 1b). Dynamic light scattering (DLS) analysis further supported this conclusion, as we noted a shift in size between 1 μM and 10 μM of PEG-PCL, indicating self-assembly of monomers into micelles at this concentration range (Fig. 1c). Furthermore, Fig. 1c shows that the size distribution of the NPs increased with increasing concentrations of PEG-PCL. To form NPs that will enter cells via endocytosis, the concentration selected was 70 μM, at which PEG-PCL NPs formed, with a mean size of 30 ± 1 nm.

### Drug loading capacity and efficiency

**Fig. 2:**
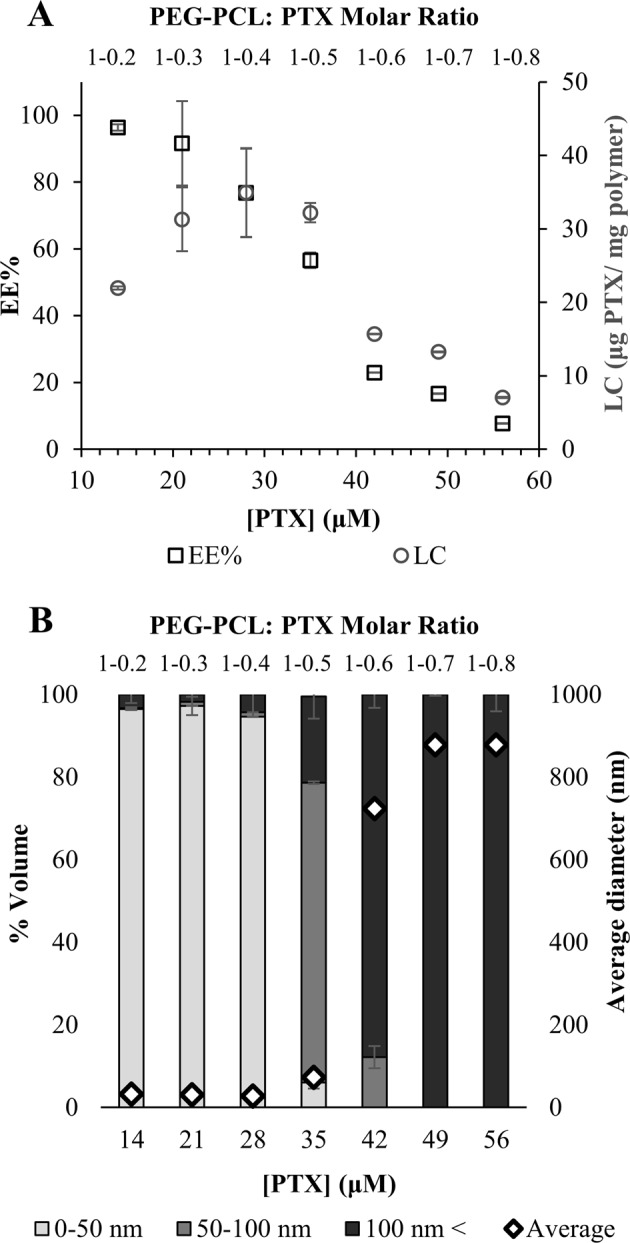
Drug loading evaluation. **a** Drug-loading capacity (○ LC) and encapsulation efficiency (□) of PTX at increasing drug: PEG-PCL molar ratio. Values presented are means ± SE. **b** NP size distribution. Volume-weighted percent of PTX in 70 μM PEG-PCL and overall average diameter (◊) as a function of the molar ratio of PEG-PCL: PTX (top axis) and drug concentration (bottom axis); error bars represent SE.

The drug loading capacity (LC) and encapsulation efficiency of PTX at increasing drug: polymer molar ratios were determined by centrifugal sedimentation of unbound excess drug and quantification of the encapsulated drug in the supernatant (Fig. 2a). At a low drug concentration range, increasing drug concentrations led to an increase in LC, up to an optimum. However, at higher drug concentrations, aggregation of free PTX was observed, as previously described^33–35^. Expectedly, the drug encapsulation efficiency decreased with increasing drug concentrations; the DLS data further support this conclusion, as the analysis reveals a dramatic increase in size between 35 μM and 42 μM of PTX (1:0.5 and 1:0.6 molar ratio of PEG: PCL and PTX), which correlates with PTX sedimentation at the same PTX concentration (Fig. 2b). Taken collectively, the formulation which displayed the maximal LC was 35 ± 6 (µg PTX/mg PEG-PCL), with an encapsulation efficiency of 77 ± 13%, and a small particle size of 32 nm, was selected for subsequent experiments.

### The morphology of PTX-loaded PEG-PCL NPs

**Fig. 3:**
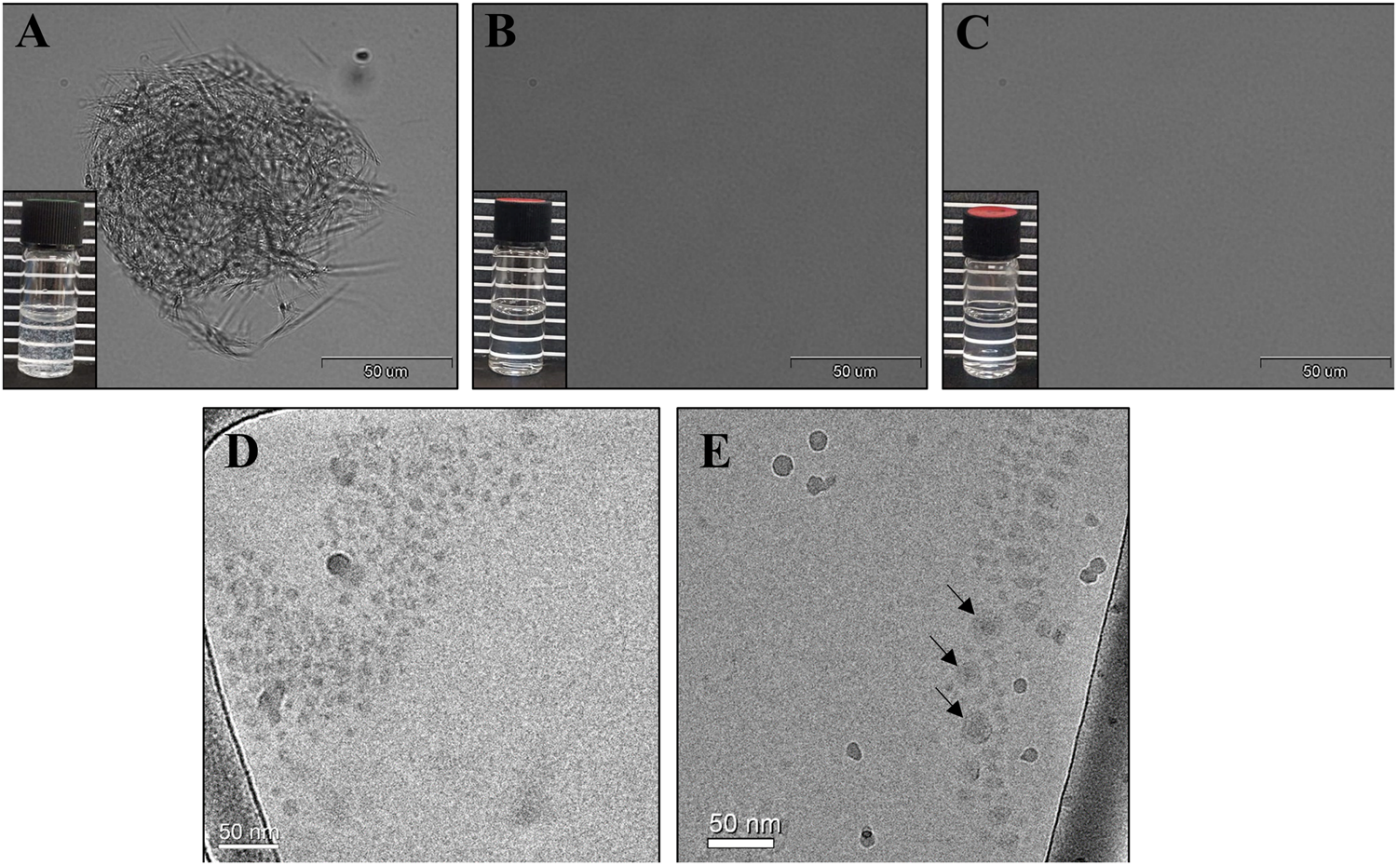
Light Microscopy observation. Light microscopy images (×60 magnification) and photographs (insets) of **a** 28 μM of PTX in water; **b** 70 μM PEG-PCL NPs in water; **c** 1:2.5 molar ratio of PTX:PEG-PCL NPs in water; Cryo-TEM images of: **d** 70 μM PEG-PCL NPs **e** 1:2.5 molar ratio of PTX:PEG-PCL NPs in water. Arrows are pointing at NPs clearly showing the core-shell structure.

The morphology of pure PTX dispersions, PEG-PCL NPs, and PEG-PCL/PTX-loaded NPs with the maximal LC in water was compared by analyzing light microscopy images, taken at a ×60 magnification using light microscopy, and the visual colloidal stability photographs (insets). Figure 3a reveals the colloidal instability of pure PTX in water that exhibits micro-sized drug crystals which tend to aggregate and precipitate, as opposed to a clear stable system in the presence of 70 μM PEG-PCL (1:2.5 PTX:PEG-PCL molar ratio) (Fig. 3c). The apparent drug solubility is significantly increased by the copolymer, resulting in clear stable solutions (or nano-dispersions), devoid of microscopic drug crystals. These results further support the association of PTX with the PEG-PCL and formation of micellar nanocapsules. Cryogenic-transmission electron microscopy (Cryo-TEM) images, at a higher magnification, were performed as shown in Fig. 3d, e. PEG-PCL micelles are apparent as round gray aggregates (Fig. 3d). The obtained size is in agreement with the DLS measurements: mean size of ~30 nm (Fig. 1c). When evaluating a 1:2.5 molar ratio of PTX:PEG-PCL NPs in water, NPs entrapping PTX were observed, with a clear core & corona structure **(**arrows, Fig. 3e).

### Conjugation of APTs to polymeric NPs

**Fig. 4:**
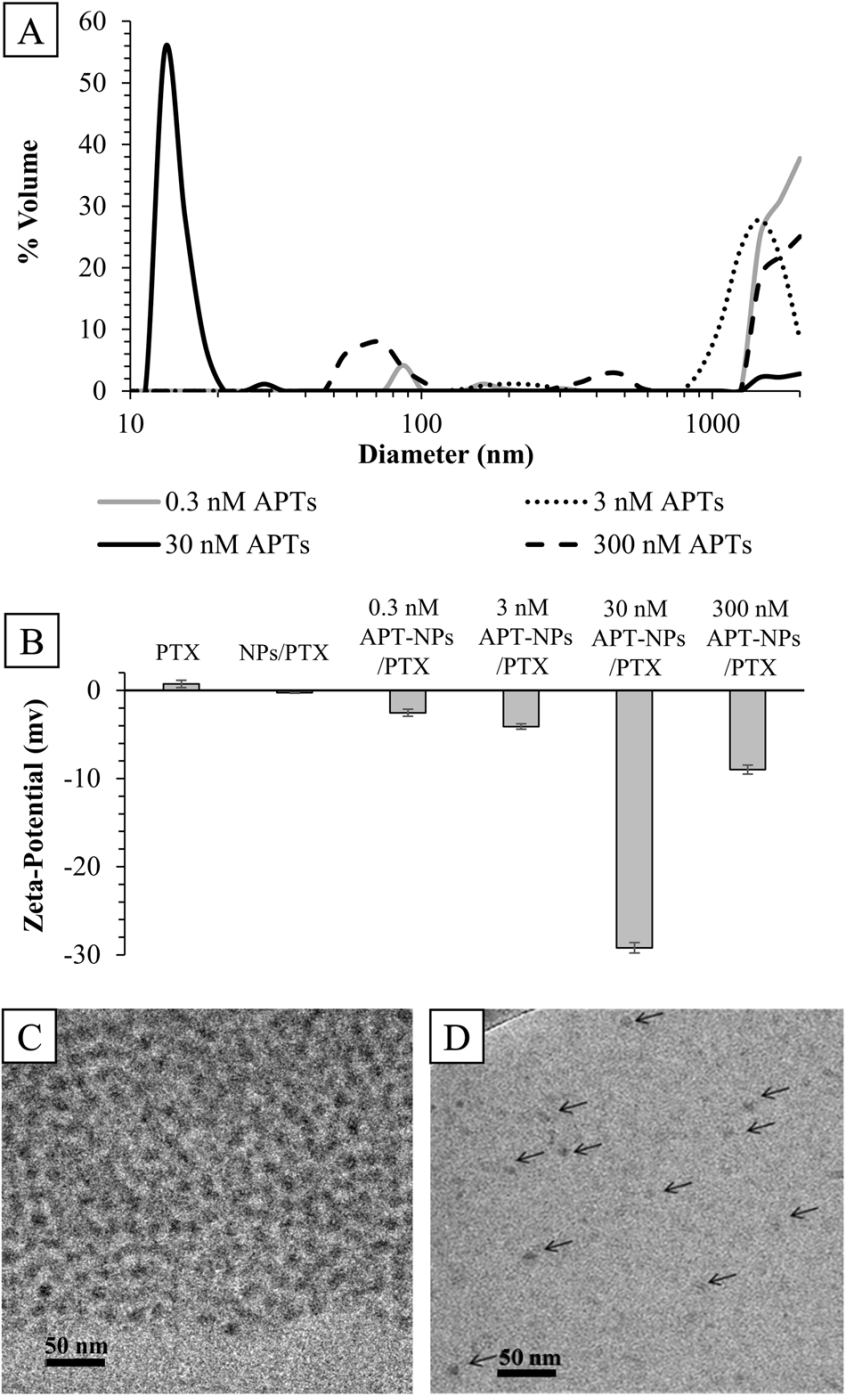
APT functionalization. **a** Particle size distribution of APT-functionalized PEG-PCL NPs, as a function of APT concentration (log scale). Mode size and percentage of bimodal distributions: APT(0.3)-NPs: 1759 nm (94%), 121 nm (6%); APT(3)-NPs: 1447 nm (95%), 210 nm (5%); APT(30)-NPs: 15 nm (93%), 1745 nm (7%); APT(300)-NPs with a trimodal size distribution of: 1750 nm (66%), 267 nm (8%), and 44 nm (26%); **b** ζ-potential values of: 28 µM PTX, 28 µM PTX encapsulated in 70 µM PEG-PCL NPs (NPs/PTX), NPs/PTX coated with 0.3, 3, 30, and 300 nM S15-APTs, respectively. Cryo-TEM images of APT-functionalized PEG-PCL NPs: **c** 0.3 nM APTs conjugated to 70 µM PEG-PCL NPs; **d** 30 nM APTs conjugated to 70 µM PEG-PCL NPs (arrows pointing to several NPs).

**Fig. 5:**
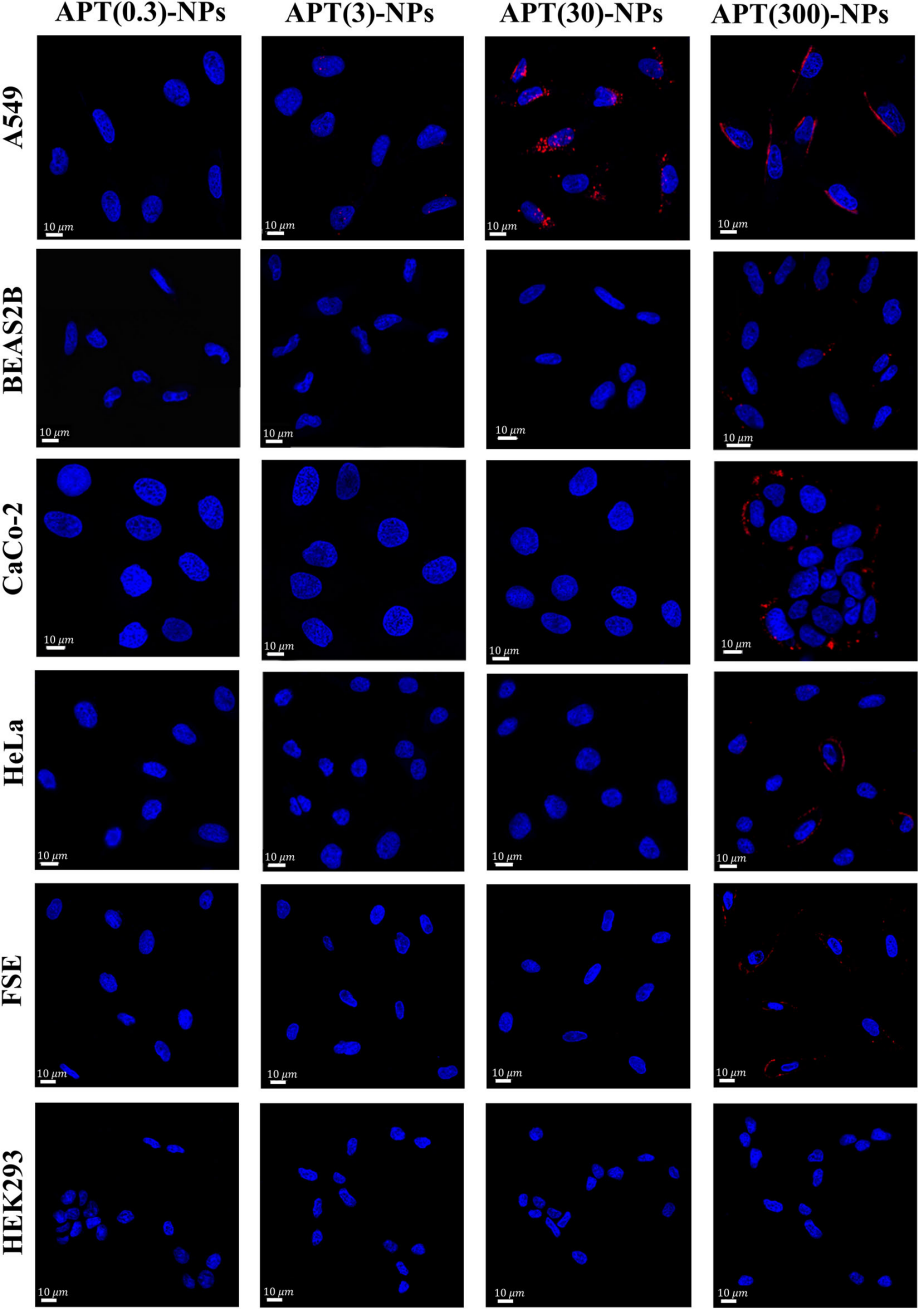
Selectivity of APT-NP internalization. Confocal laser microscopy images of cells exposed to PEG-PCL NPs decorated with increasing concentrations of APTs. A549, BEAS2B, CaCo-2, HeLa, FSE, and HEK-293 cells were studied. Samples were diluted 1:5 (v/v) in FBS-free medium and incubated with varying APT concentrations for 2 h at 37 °C. APTs were labeled with Cy-5 (red). Nuclear DNA was labeled with Hoechst 33342 (2 μg/ml) (blue).

To generate NPs that will selectively target NSCLC, S15-APTs were covalently conjugated to polymeric NPs. Amine-modified S15-APTs were conjugated to a carboxyl group on the PEG to form an amide linkage. The optimal amount of APT conjugated to the NPs was determined by minimizing particle size, and by maximizing both the specificity to target cells and the diagnostic fluorescence signal for tumor cell localization. The samples presented in Figs. 4 and 5 were prepared as elaborated in section 5.2.6. As the APT:NPs ratio increased, the NP size decreased down to a bimodal size distribution with an average size of 15 nm for 93% of the NPs, and 1745 nm for 7% of the NPs at 30 nM APT conjugated to 70 µM PEG-PCL NPs (APT(30)-NPs), as supported by both DLS and cryo-TEM. These NPs showed the most negative ζ-potential of −29.2 ± 0.6 mV (Fig. 4), hence were stable. At lower APT concentrations (0.3 and 3 nM APTs termed APT(0.3)-NPs and APT(3)-NPs), the NPs tended to aggregate over time. At the highest APT concentration of 300 nM (APT(300)-NPs), the NP size increased again.

The internalization of APT-NPs by A549 cells was compared to that of normal human bronchial epithelial BEAS2B, cervical carcinoma HeLa, colon adenocarcinoma CaCo-2, neonatal foreskin fibroblast FSE, and human embryonic kidney HEK-293 cells, using confocal laser microscopy (Fig. 5). The samples were diluted 1:5 (v/v) in fetal calf serum (FCS)-free medium. Following 2 h incubation with increasing APT concentrations at 37 °C, A549 cells incubated with 14 µM APT(30)-NPs displayed internalization, as revealed by the red fluorescent Cy5-labeled-APTs, which accumulated in endosomes, as seen by the red fluorescent intracellular vesicles (Fig. 5). In contrast, neither normal BEAS2B, FSE, and HEK-293 cells, nor malignant HeLa and CaCo-2 cells displayed any cellular fluorescence when exposed to 14 µM APT (30)-NPs. These results confirm that S15-APTs retained their selectivity to target NSCLC cells after being conjugated to the NPs. In the presence of the APT(0.3)-NPs, and APT(3)-NPs, almost no internalization into target A549 cells was observed. Increasing APT concentrations during NP decoration resulted in enhanced cell binding and fluorescence signal. Interestingly, at a high APT concentration of 300 nM during decoration, APT-decorated NPs adsorbed to cell lines in an unspecific manner. Thus, the APT-NPs selected for further studies were those decorated at 30 nM APT, as they maintained both a small particle size, and a very negative ζ-potential, resulting in colloidally stable NPs, while retaining the specificity to target NSCLC A549 cells (Figs. 4 and 5).

### In vitro drug release

**Fig. 6:**
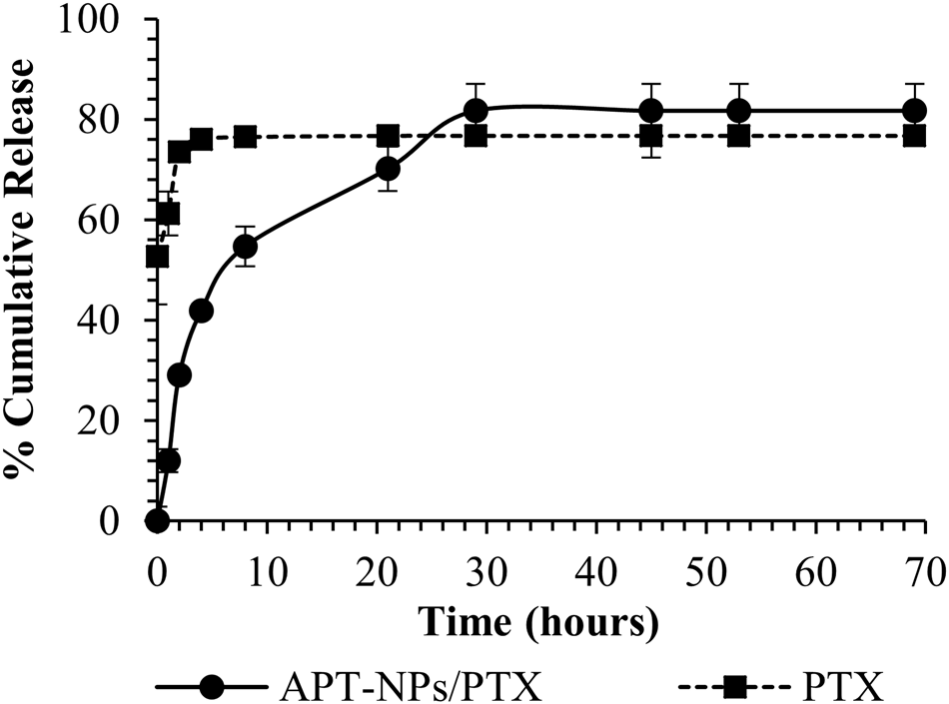
Drug Release kinetics. In vitro (cell free) drug release profile of PTX-loaded APT-NPs (black line) compared to unencapsulated PTX (dashed line). One ml of APT-NPs/PTX or PTX was suspended in 30 mL of PBS containing 0.1% Tween 80 and incubated at pH 7.4, 37 °C with agitation. At designated time points, the 30 ml PBS containing Tween 80 was replaced by fresh buffer. The samples were freeze-dried and then dissolved in ACN. The amount of PTX at each time point was quantified by HPLC. Data represent means ± SE, *n* = 3.

The in vitro release of PTX from APT (30)-NPs was assessed by dialysis in a large volume of phosphate buffer solution (10 mM phosphate, pH 7.4) containing Tween 80 (0.1% wt) at 37 °C to capture the released PTX by the non-ionic detergent^36^. At each time point, the entire dialysis buffer solution was replaced by a fresh buffer. The release of PTX from the APT-NPs with time is depicted in Fig. 6; PTX was released from the polymeric NPs after 30 h in a sustained release manner, as opposed to a burst release of unencapsulated drug.

### Clathrin-mediated endocytosis is the mechanism underlying internalization of APT-NPs into A549 cells

**Fig. 7:**
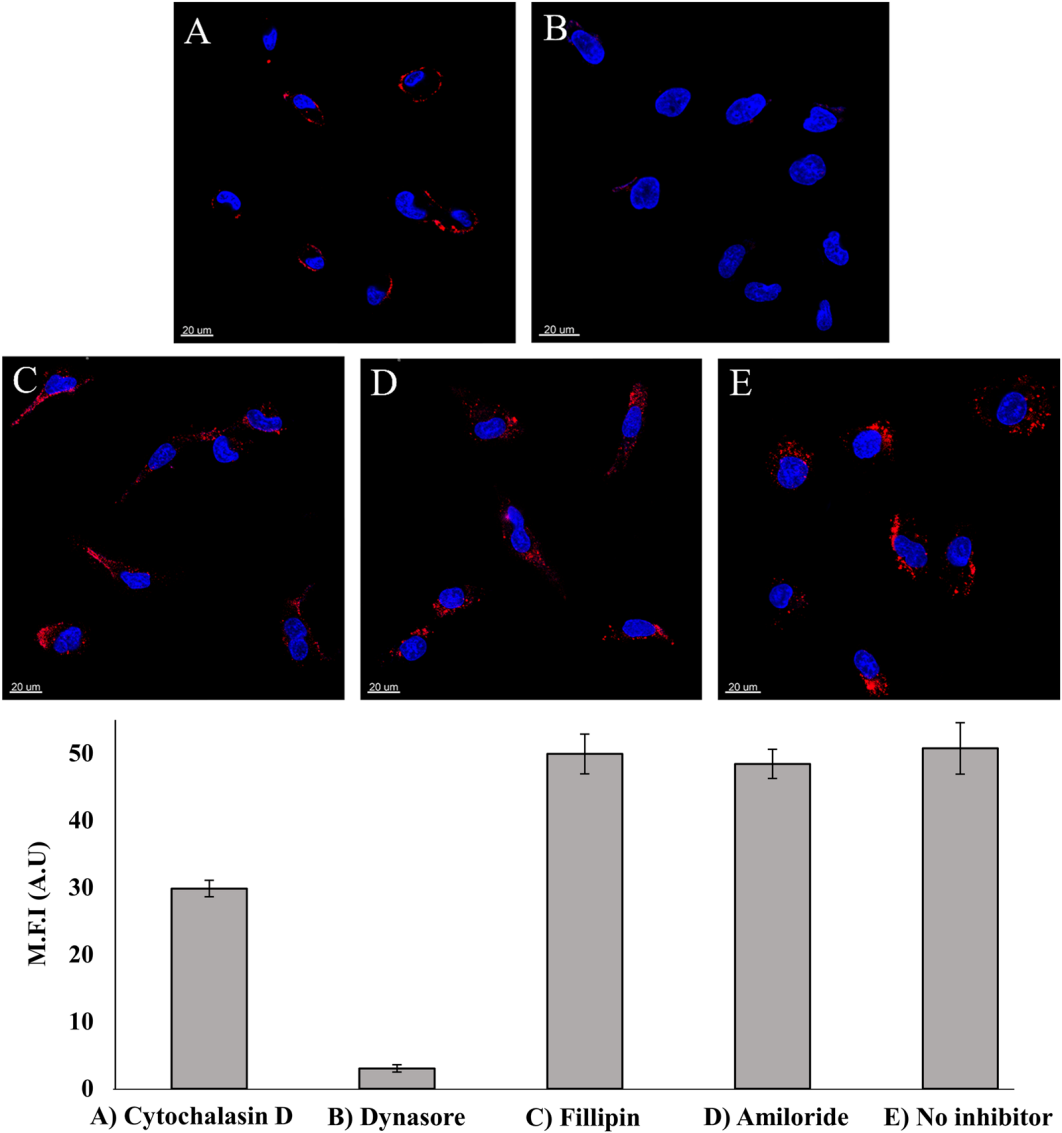
Disruption of endocytosis by pre-incubation of A549 cells with different inhibitors. Top: **a** 10 μM Cytochalasin D for 30 min; **b** 80 μM Dynasore for 30 min; **c** 1 mM Amiloride for 10 min; **d** 1 μg/ml Filipin for 30 min; and **e** drug-free medium; followed by incubation with 14 µM APT (30)-NPs for 2 h. Nuclear DNA was labeled with Hoechst 33342; *Bottom:* Mean fluorescence intensity (M.F.I) values of APT(30)-NPs in A549 cells incubated with different inhibitors were determined with IMARIS software for analysis of image data. The red fluorescence channel was defined between 10 and 100 for all images presented. Values presented are means ± SE.

We next undertook experiments to verify that the uptake of APT-NPs proceeds via endocytosis, as we previously found for quantum dots (QDs)-labeled APTs^37^. We used inhibitors of several processes related to endocytosis. First, we determined the involvement of the actin cytoskeleton, which takes an active role in regulating endocytosis by trafficking endosomes along the cytoskeleton^38^. A549 cells were pretreated with the established actin polymerization inhibitor, cytochalasin D, which disrupts endocytosis. A549 cells were studied by confocal laser microscopy for the uptake of APT(30)-NPs (Fig. 7); cells exposed to cytochalasin D displayed a marked decrease in the uptake of APT-NPs, consistent with an endocytic internalization. Moreover, it is apparent that the APT-NPs were bound to the surface of these cytochalasin D-treated cells without undergoing internalization due to cytoskeleton disruption (Fig. 7a). We thus further investigated the pathway responsible for the receptor-mediated endocytosis. A549 target cells were subjected to various established endocytosis inhibitors and analyzed by confocal laser microscopy (Fig. 7b–d)^37^. Neither pretreatment with filipin (Fig. 7c), an inhibitor of caveolae-mediated endocytosis, nor with amiloride (Fig. 7d), an inhibitor of pinocytosis, inhibited APT-NPs internalization by A549 cells. The red fluorescence intensity of Cy5-labeled APT-NPs in the presence of filipin or amiloride was similar to that of the drug-free control (Fig. 7e). In contrast, Cy5-labeled APT-NPs internalization was abolished upon pretreatment with the established dynamin inhibitor, Dynasore, which blocks clathrin-mediated endocytosis (CME) (Fig. 7b). Hence, the mechanism underlying internalization of APT-NPs is CME.

### Selective APT (30)-NPs cytotoxicity to A549 cells

**Fig. 8:**
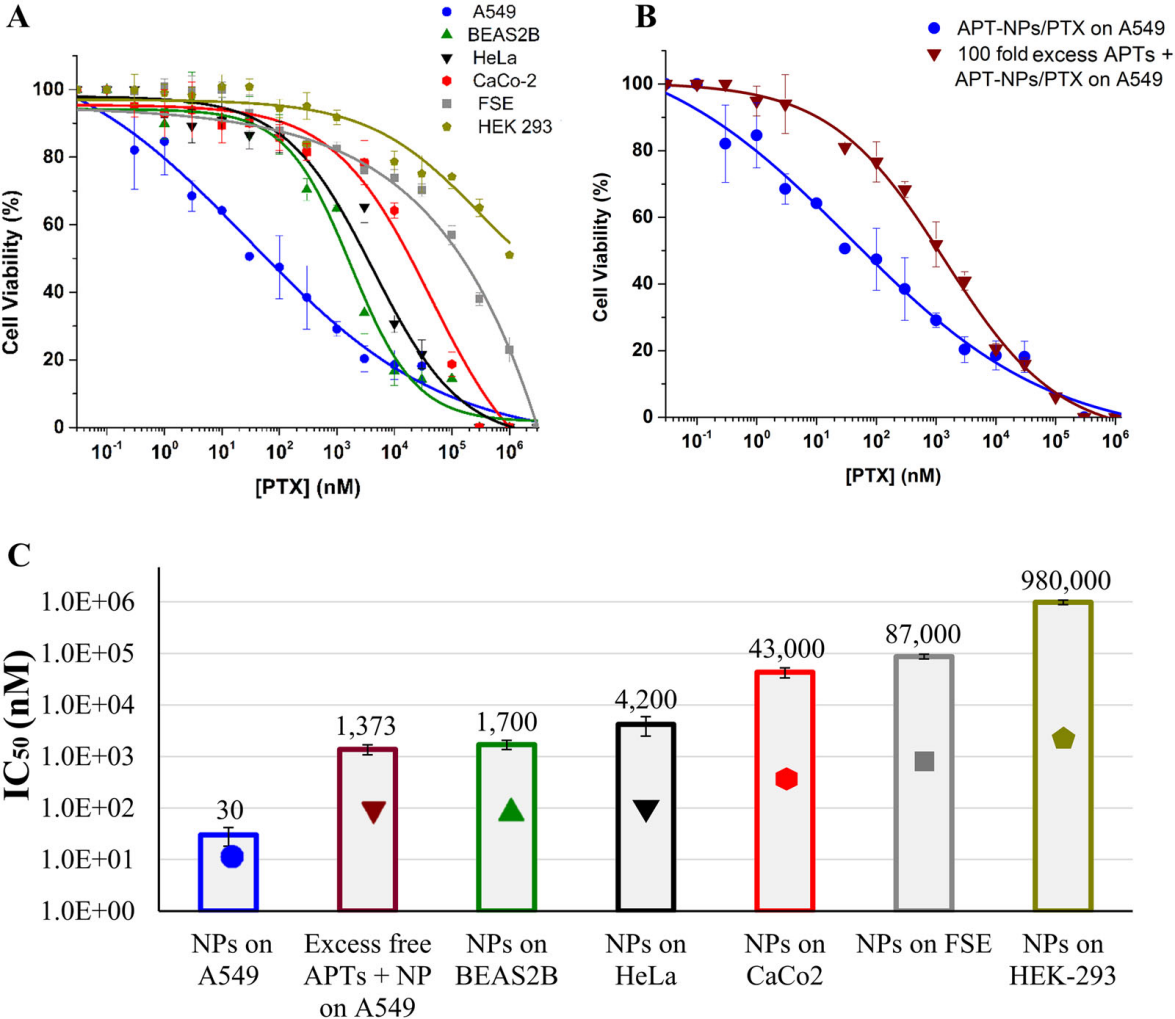
Selective cytotoxicity to A549 target cells. Cell viability as a function of PTX concentration of: **a** APT-NPs entrapping PTX (2.5:1 molar ratio) were added to A549 target cells and to BEAS2B, HeLa, CaCo-2, FSE, and HEK-293 cells; **b** APT-NPs entrapping PTX (2.5:1 molar ratio) added to A549 target cells vs. competitive binding conditions using a 100-fold excess of free S15-APTs, co-incubated with APT-NPs entrapping PTX. Values presented are means ± SE. Sigmoidal model curve were fitted using Eq. (3); **c** IC_50_ values derived from the fitted dose-response curves; *P*-values obtained were <0.01 for BEAS2B and HeLa cells, <0.001 for CaCo2 and HEK-293 cells, and <0.0001 for FSE cells. (Symbols marked on the bars are only for identification).

The selective cytotoxicity of PTX-loaded APT(30)-NPs towards A549 cells was studied (Fig. 8a). These APT (30)-NPs displayed IC_50_ values of 0.03 µM against target A549 cells, and 1.7, 4.2, 43, 87, and 980 µM PTX towards BEAS2B, HeLa, CaCo2, FSE, and HEK-293 cells, respectively (Fig. 8c). To further corroborate that the specificity of NPs towards A549 cells is attributable to the S15-APTs, PTX-loaded APT(30)-NPs were co-incubated with a 100-fold excess of free S15-APTs; a complete competitive abolishment of S15-APTs binding to A549 cells was observed, suggesting that the binding and internalization of these NPs is mediated by the S15-APTs (Fig. 8b).

## Discussion

Particle size is a design parameter of paramount importance, as it affects the capacity of endocytic uptake by tumor cells, the accumulation of NPs within tumors via the EPR effect, as well as elimination by the RES. It was previously demonstrated that only NPs <50 nm in size are able to accumulate in poorly permeable tumors^39^. Hence the most efficient uptake occurs for 10–50 nm NPs^20,22–24^. To this end, we herein aimed at developing 10–50 nm NPs. Previously described polymeric NP systems were mostly >100 nm^20,40–42^. By using a short block-copolymer with a molecular weight ratio of 1:2 (2.5 KDa:5 KDa) between the PCL and PEG, respectively, we were able to form small NPs with a diameter of 30 nm with a spherical structure due to a small packing parameter (Fig. 1a). The CMC revealed the thermodynamic stability, which is an important parameter reflecting the utility of the nanocarrier platform (Fig. 1b). Another important design feature to minimize opsonization is a hydrophilic particle surface which was achieved by PEGylation. The CMC of our PEG-PCL was consistent with those of other pegylated amphiphilic copolymers^20^. The obtained size and the formation of a micelle structure was confirmed by DLS measurements, and is in accord with the cryo-TEM images (Figs. 1c and 3d).

The selected formulation with the maximal LC of 35 ± 6 (µg PTX/mg PEG-PCL), and encapsulation efficiency of 77 ± 13%, outperforms many previously reported systems, where the encapsulation efficiency ranged between 40 and 60% with lower drug loading^33,43^. PTX-loaded NPs were observed by cryo-TEM, further supporting the drug encapsulation inside the micelles (Fig. 3e). The drug release profile was in agreement with polymer based NPs platforms^44^, depicting an initial burst release (presumably of the small unencapsulated drug fraction) followed by a sustained release of the drug entrapped by the polymeric chains. In our experiments, the complete replacement with fresh buffer caused a drug gradient much higher than in other release experiments in the literature, causing a stronger release driving force (Fig. 6). Nevertheless, when comparing to an APT-decorated PLGA NP system encapsulating PTX, drug released into a detergent-free buffer (i.e lacking Tween 80 which favors drug release), our NP system displayed an equal sustained release profile^45^. Moreover, the drug release is influenced by the length of the core-forming polymer segment^46^. When comparing our 5 KDa hydrhophobic core to the average M.W. of 50 KDa of the hydrophobic core of PLGA previously reported^45^, the drug release behavior was not different. These results suggest that due to the higher hydrophobicity of PCL compared to that of PLGA, the stronger attraction between PCL and PTX allowed for the use of a shorter polymer block length, which was also important for forming small NPs (<50 nm), as there is a direct relation between the hydrophobic block length and particle size^38^.

The optimal amount of APTs conjugated to the NPs was determined. After sample preparation, NPs were incubated for 2 additional hours to attain equilibrium. At low APT concentrations (0.3 and 3 nM), the NPs tended to aggregate over time as determined by DLS, ζ-potential, and cryo-TEM analysis (Figs. 4a–c); these data are consistent with a previous study demonstrating decreasing aggregation with increasing APT density on NPs^47^. At these low APT concentrations, essentially no internalization was observed in target A549 cells, as studied by confocal laser microscopy (Fig. 5). This result is expected as aggregation of NPs inhibits their internalization. As the APT:NPs ratio increased, smaller NPs formed, up to a minimal size of 15 nm for 93% of the APT(30)-NPs population (Fig. 4a). The small NP size obtained could be explained by the highly negative charge of the phosphate groups of the nucleotide-based APTs, causing repulsion between the negatively charged NPs having a very negative ζ-potential, and contributing to the stability and small size of the NP system, as also revealed by the ζ-potential and cryo-TEM analyses (Fig. 4b, d). Following APTs conjugation, a decrease in size (from 30 nm to 15 nm) was observed due to the electrostatic repulsion forces between APT(30)-NPs. Furthermore, the repulsion effect is more significant in pure water than in buffer solution, as the buffer ions diminish inter-particle repulsion by screening of the APTs charge. This selected formulation of NPs formed by decoration at a concentration of 30 nM APT, also maintained the specificity to A549 target cells, as evident from the red fluorescent APTs (labeled with Cy-5) that apparently accumulated in endosomes visible as red intracellular vesicles (Fig. 5). In contrast, neither normal BEAS2B cells, nor HeLa and CaCo-2 cells, or FSE and HEK-293 cells showed any cellular fluorescence when exposed to the APT (30)-NPs, confirming that the S15-APTs maintained their specificity and affinity to A549 target cells after being conjugated to the NPs, evidencing the successful synthesis.

It was previously shown that with rising APT concentrations, enhanced binding and fluorescence signal were obtained^48^. Here we observed this trend only up to 30 nM APT conjugated to 70 µM PEG-PCL. However, at the highest APT concentration of 300 nM, the size of the NPs increased (Fig. 4a). This could be explained by the high APT density on the surface of NPs, which may have led to an extended APT conformation due to molecular crowding, which interferes with the ability of APTs to fold into a distinct tertiary structure that endows them with the specificity to target NSCLC cells. The unfolded conformation may have led to inter-particle APT complementation and consequent aggregation. This hypothesis was further supported by ζ-potential measurements and confocal laser microscopy. As the APT concentration used in the conjugation to the NPs increased, a more negative ζ-potential was obtained, as expected, up to a minimal value (−29.2 ± 0.6 mv) for APT(30)-NPs. At higher concentrations of 300 nM APT, there was a substantial increase in the ζ-potential value (−8.98 ± 0.5 mV; Fig. 4b), further supporting the formation of inter-NP interactions among APTs that were not able to fold properly due to their high density, resulting in NPs instability and aggregation. Confocal laser microscopy shows that for APT(300)-NPs, no internalization occurred (Fig. 5), and apparently, unspecific adsorption to positive areas on the surface of plasma membrane (e.g. sphingosine, or positively charged patches on membrane proteins) can be observed, resulting from high APT density. Another advantage for not using the highest APT density on the NP is to avoid unnecessary masking of PEG on the NPs surface by excess of APTs, as the exposure of PEG contributes to anti-biofouling properties^42^ and stealth properties for prolonged circulation time.

We found (Fig. 7) that APT(30)-NPs are taken up via classical clathrin-mediated endocytosis (CME), as we have recently reported for S15-APT-decorated QDs that possess a smaller size than the APT-PEG-PCL NPs^37^. In the clinical setting, it is expected that internalization via CME will enable an efficient cellular uptake of PTX-loaded APT-NPs and achieve targeted drug delivery, resulting in efficacious therapeutic treatment. Moreover, uptake via CME facilitates enhanced cellular uptake and intracellular trafficking^49^. The spherical APT-NPs (Fig. 4d) formed at an APT concentration of 30 nM, have a suitable size (15 nm) for CME^23^, while it was also demonstrated previously that spherical particles are able to undergo faster internalization than non-spherical NPs^50^. Internalization of APT-NPs via CME proceeds via endosomes that eventually fuse with lysosomes and are degraded by hydrolytic enzymes therein^51^. The phosphodiester bond between two nucleotide residues in the APTs is cleaved by endo/exo-nucleases^52^, whereas the ester bonds in PCL are cleaved by lipases^53^. Consequently, the hydrophobic drug PTX will be effectively released, hence diffusing to the cytoplasm.

Our results demonstrate selective cytotoxicity of the PTX-loaded NPs against target A549 cells, further corroborating the confocal laser microscopy results. APT(30)-NPs entrapping PTX displayed half maximal inhibitory concentration (IC_50_) value of 0.03 µM against A549 cells, while revealing a 57-, 140-, 1438-, 2900-, and 32,667-fold higher IC_50_ values for non-target cell lines (BEAS2B, HeLa, CaCo-2, FSE, and HEK-293) respectively; (Fig. 8a, c). While all of these cytotoxicity comparisons between the target cells and the different non-target cells showed high selectivity to the target cells (as apparently the target A549 cancer cells presumably overexpress a certain receptor protein to which the aptamer selectively binds), the differences between the extent of selective cytotoxicity can be explained as follows: the lowest selectivity ratio (57-fold) was that compared to the healthy bronchial cell line, and it can be explained by the fact that these cells originate from the same tissue lineage, hence are most similar to the target cells. Higher selectivity ratios of 140- and 1438-fold were obtained when comparing A549 to HeLa and to CaCo-2 cells, respectively. Both of these latter cell lines are malignant cells which might have a slightly higher molecular and phenotypic resemblance to A549 than the healthy cell lines FSE and HEK-293, which are not related to the lung cell lineage. Hence, in the latter two cell lines, striking 2900-fold and 32,667-fold selectivity ratios were obtained compared to A549 cells. These preclinical cytotoxicity results suggest an outstanding therapeutic window that outperforms existing APT-targeted NPs by 10-fold^54,55^. To confirm that this remarkable cytotoxic selectivity to A549 cells is attributable to the S15-APTs, a competition with a 100-fold molar excess of free S15-APTs was performed (Fig. 8b). A two orders of magnitude shift in the IC_50_ values demonstrated that the S15-APT component accounts for NPs binding to target A549 cells. These results indicate that the internalization of the S15-APT-decorated NPs is APT-dependent and that APTs are not toxic to the cells even at high concentrations.

Furthermore, the specificity of S15-APT was explored previously by our group in human cell lines^37^. Hence, these APT-NPs hold great promise towards eradication of NSCLC cells. It should be noted that chemotherapeutic agents including PTX inflict bone marrow suppression and consequent neutropenia. Inasmuch as we have not studied bone marrow-derived cells, future studies are warranted to evaluate the impact of PTX on bone marrow-derived cells in vivo. Studies are underway to assess the anti-tumor activity of these APT-NPs in mice bearing A549 human xenografts.

## Conclusions

A delivery system comprising a biocompatible block-copolymer, PEG-PCL, entrapping the hydrophobic chemotherapeutic drug PTX within its micelles, was developed. The dual utility of aptamer-decorated NPs for both drug stabilization and tumor targeting was studied and the optimal aptamer density on the NPs was determined. PTX-loaded APT (30)-NPs were approximately 15 nm in size (i.e. within the ideal size range for cancer cell targeting: 10–50 nm), they were colloidally stable, and exhibited specific targeting of A549 cancer cells. These APT-NPs demonstrated efficient encapsulation of PTX, high specificity to- and potent eradication of, human NSCLC A549 cells. Hence, these novel findings have promising implications for the development of NPs targeted by S15-APTs, for both drug delivery and tumor diagnostics. This remarkable therapeutic window may facilitate the administration of minimal drug doses, while enhancing the efficacy of the chemotherapeutic drug treatment, reducing side effects, and the cost of treatment.

## Materials and methods

### Materials

All chemicals were obtained from Sigma-Aldrich (Merck) (Rehovot, Israel) unless otherwise stated. Polyethylene glycol (PEG) (5KDa): polycaprolactone (PCL) (2.5 KDa) block-copolymer containing a carboxylic end group at the PEG terminus was custom-synthesized (Creative PEGWorks, Durham, NC). Cy5-labeled S15-APTs harboring the following nucleotide sequence: 5′- Cy5-ACG CTC GGA TGC CAC TAC AGG CTA TCT TAT GGA AAT TTC GTG TAG GGT TTG GTG TGG CGG GGC TAC TCA TGG ACG TGC TGG TGA C-3′^11^ were purchased from BioSpring Biotechnolgy GmbH, (Frankfurt, Germany). 1,6-diphenyl-1,3,5-hexatriene (DPH) was kindly provided by Associate Prof. Boaz Mizrahi, Faculty of Biotechnology and Food Engineering, Technion, Israel.

### Methods

#### Preparation of PEG-PCL NPs and their critical micelle concentration (CMC)

Self-assembled PEG-PCL micelles were prepared by surfactant-free nanoprecipitation with minor modifications of a previous protocol^32^. PEG-PCL was dissolved in acetonitrile (ACN); ACN was then added dropwise into ultrapure water (Biological Industries, Kibbutz Beit-HaEmek, Israel) at a ratio of 1:2 (v/v), and stirred until complete solvent evaporation of ACN was obtained. The CMC of the polymer was determined by enhancement of the absorbance of the hydrophobic dye DPH upon entrapment within micelles. The PEG-PCL solution was prepared at various concentrations. Then, DPH (dissolved in ethanol) was added at a final concentration of 4 μM. The relative absorbance at 378 nm/400 nm was plotted against the PEG-PCL concentration, and the point at which the two lines intersect was defined as the CMC of the copolymer^56^.

#### Particle size distribution and zeta-potential analyses

Volume-weighted particle size distributions of the polymeric self-assembled micelles and micelles encapsulating PTX at different PEG-PCL to drug molar ratios were studied using a dynamic light scattering (DLS) analyzer, NICOMP™ Particle Sizing System (Santa Barbara, CA, USA) as previously described^57^. Electrophoretic mobility was determined using a Zetasizer Nano instrument (Malvern Instruments Ltd., Worcestershire, UK). Zeta potential was derived based on the Smoluchowski model. Samples were prepared by the nanoprecipitation method as described above. PTX was co-dissolved with PEG-PCL in ACN. Then, the ACN solution was added dropwise to water and stirred in a hood until complete evaporation of the organic solvent was obtained. Samples were left for 2 additional hours to equilibrate. Measurements were made in triplicates. Mean values and standard error (SE) were calculated.

#### Analysis of drug loading capacity (LC) and encapsulation efficiency (EE)

To quantify the amount of drug loaded into the PEG-PCL NPs [LC (µg PTX/ mg polymer)] and the EE (%) of PTX, samples of 70 µM PEG-PCL (0.525 mg/ml) containing PTX at increasing molar ratios of drug:PEG-PCL, were prepared. Samples were centrifuged at 10,000 × *g* at 4 °C for 20 min to sediment the unbound excess drug aggregates. Quantification of the encapsulated drug in the supernatant was performed by lyophilizing the supernatant and dissolving it in ACN to extract the drug from the micelles. The concentration of PTX was determined by HPLC as previously described^58^. Samples were analyzed using a linear calibration curve. Results are presented as means ± SE of two independent experiments, each performed in duplicates.

Calculation of the loading capacity (LC) and encapsulation efficiency (EE) of PTX in PEG-PCL micelles was performed using Eqs. (1) and (2)^59^:

1 $${\mathrm{LC}}\left( {\frac{{{{\upmu}} {\mathrm{g}}_{{\mathrm{PTX}}}}}{{{\mathrm{mg}}_{{\mathrm{PEG}} - {\mathrm{PCL}}}}}} \right) = \frac{{W_{{\mathrm{ED}}}}}{{W_{{\mathrm{PEG}} - {\mathrm{PCL}}}}}$$

2 $${\mathrm{EE}}\left( {\mathrm{\% }} \right) = \frac{{W_{{\mathrm{ED}}}}}{{W_{{\mathrm{TD}}}}} \times 100$$

Where *W*_ED_ is the amount of encapsulated drug, *W*_PEG−PCL_ is the amount of polymer in the sample, and *W*_TD_ is the total amount of drug in the sample.

#### Morphological studies

##### Light microscopy

The morphology of PTX crystal formation in the presence or absence of PEG-PCL was studied by light microscopy (Olympus DP71 digital camera connected to an Olympus BX51 microscope) (×60 magnification at a temperature of 24 °C). Samples of 70 µM PEG-PCL, 28 µM pure PTX, and 70 µM PEG-PCL encapsulating 28 µM PTX, were prepared by dissolving PTX in ACN and adding it dropwise to water at a ratio of 1:2 (v/v), while stirring until complete ACN evaporation was obtained. The samples were left for 2 h to equilibrate and were evaluated by light microscopy. Ten images of each sample were collected in three independent experiments.

##### Cryogenic-transmission electron microscopy imaging

Cryo-TEM (Philips CM120 microscope) analysis was used for the imaging of PEG-PCL NPs, and PTX was entrapped in PEG-PCL NPs at a 1:2.5 molar ratio. Samples were prepared as described for light microscopy imaging. Specimen preparation and evaluation were previously described^60,61^. Gatan Multi Scan 791 cooled CCD camera was used to acquire the images, using the Digital Micrograph 3.1 software package.

#### Cell cultures

Human NSCLC A549, HeLa cervical carcinoma, normal human bronchial epithelial BEAS2B cells, and human embryonic kidney HEK-293 cells were cultured in RPMI-1640 medium, supplemented with 10% fetal bovine serum (FBS), 2 mM glutamine, 100 μg/ml penicillin and streptomycin (Biological Industries, Beit-HaEmek, Israel). CaCo-2 cells and neonatal foreskin fibroblast FSE cells were maintained in DMEM medium, supplemented with 10% FBS, 2 mM glutamine, 100 μg/ml penicillin and streptomycin (Biological Industries, Beit- Haemek, Israel). Cells were incubated at 37 °C in a humidified atmosphere of 5% CO_2_. BEAS2B cells and FSE cells were generously provided by Prof. Rotem Karni (The Hebrew University, Jerusalem, Israel), and Prof. Ami Aronheim (Technion, Haifa, Israel), respectively.

#### Aptamer functionalization and characterization of NPs specificity by confocal laser microscopy

S15-APTs were covalently conjugated to polymeric NPs; amine-modified S15-APTs were conjugated to a carboxyl group on the PEG to form an amide linkage, using the well-established N-hydroxysuccinimide (NHS), EDC (1-ethyl-3-(3-dimethylaminopropyl)-carbodiimide) conjugation strategy as previously described^43^. Polymeric NPs were coupled to APTs for 2 h, ultra-filtered three times with 100 KDa MWCO (Merck, Darmstadt, Germany) to remove unreacted APTs and left unstirred for 2 additional h to equilibrate; PEG-PCL NPs “decorated” with APTs were termed APT-NPs.

Selective internalization of APT-NPs, as a function of APT concentration was studied in A549, BEAS2B, HeLa, CaCo-2, FSE, and HEK293 cells. Cells were seeded on μ-slides VI 0.1 (Ibidi, Martinsried, Germany) at 50% confluence and incubated overnight to allow for cell attachment. Cells were then washed with phosphate buffered saline (PBS); for internalization studies, APT-NPs were diluted 1:5 (v/v) in FBS-free medium, and incubated for 2 h at 37 °C. Cells were washed three times with PBS to remove unbound APT-NPs. Cells were then incubated with 2 μg/ml Hoechst 33342 in growth medium for 10 min to achieve nuclear DNA staining. Fluorescence was studied using an inverted confocal microscope (Zeiss LSM 710). Two fluorescence channels were used during all image capturing: Blue for the viable DNA-dye Hoechst 33342 and red for the Cy5 dye. The method for determining the impact of various internalization inhibitors on APT-NPs uptake was described in a recent study^37^. APT-NPs were diluted 1:5 (v/v) in FBS-free medium, and incubated for 2 h at 37 °C. APT-NPs were covalently labeled to Cy5 to allow fluorescent tracking.

#### In vitro drug release

The release profiles of PTX from APT-NPs and free PTX were investigated using the dialysis method. One mL of PTX-loaded APT-NPs or free PTX (both systems in water) were placed in dialysis bags (molecular weight cutoff = 3500 Da), (Sigma-Aldrich, Merck, Rehovot, Israel) which were then incubated in 30 mL phosphate buffer (10 mM phosphate, pH 7.4) containing Tween 80 (0.1% wt) at 37 °C with gentle shaking^36^. At given time points (0, 1, 2, 4, 8, 21, 29, 45, 53, and 69 h) the dialysis medium was collected and replaced by the same volume (30 ml) of fresh buffer containing Tween 80; the latter was performed in order to have sufficient PTX for quantification (above the HPLC quantification limit). To calculate the cumulative amount of PTX released, samples from each time point were freeze-dried, dissolved in ACN, and analyzed by HPLC. Results presented are means of three experiments ± SE.

#### Cytotoxicity assays

The selective cytotoxicity of PTX entrapped in APT-NPs was studied in A549 target cells, and in BEAS2B, HeLa, CaCo-2, FSE, and HEK-293 non-target cells. Moreover, the cytotoxicity of APT-NPs entrapping PTX towards target A549 cells was evaluated while co-incubating these cells with a 100-fold molar excess of free APTs. The preparation of PTX-loaded APT-NPs is described in the “Particles size distribution analysis by DLS” section. Non-encapsulated PTX was prepared using the same protocol, in the absence of the polymer.

A549, HeLa, BEAS2B, CaCo2, FSE, and HEK-293 cells were seeded at 0.6 × 10^4^, 1 × 10^4^, 2 × 10^4^, 4 × 10^4^, 2 × 10^4^, and 3 × 10^4^ cells/ml, respectively, in 96-well plates. Following a 24-h incubation, cells were exposed to the APT-NPs/PTX at increasing PTX concentrations (0.1 nM–0.1 mM) for 2 h, followed by three washes with growth medium. After an additional 72 h incubation to allow the drug to elicit its cytotoxic activity, cell growth inhibition was determined using a colorimetric XTT-based cell proliferation assay (Biological Industries, Beit-HaEmek, Israel). Cells grown in a drug-free medium or with added 70 µM PEG-PCL, and 70 µM APT-PEG-PCL, served as the controls. Results shown are means ± SE obtained from three independent experiments, each performed in triplicates.

The data were analyzed using a nonlinear curve fitting of a sigmoidal model (Hill1) with OriginPro 9.0 for dose-response curve according to Eq. (3)^59^:

3 $${{P}} = {{P}}_\infty + ({{P}}_0 - {{P}}_\infty ) \times \left( {\frac{{[{{D}}]^{{n}}}}{{{{{\mathrm{IC}}}}_{50}^n + [{{D}}]^{{n}}}}} \right)$$

*P* stands for the percentage of live cells; *P*_∞_ represents the minimal percent of live cells at infinite drug concentration (zero cell viability); *P*_0_ is the maximal percent of surviving cells in the absence of drug (100%=control); [*D*] stands for the drug concentration; IC_50_ stands for the drug concentration exerting 50% inhibition of cell growth; and *n* is the Hill slope parameter for the abruptness of the dose-response curve. The statistical analysis of variance of the calculated IC_50_ values was determined using an unpaired student’s *t*-test. A *P*-value < 0.01 was considered statistically significant.

## References

[1] Torre Lindsey A., Siegel Rebecca L., Jemal Ahmedin (2015). Lung Cancer Statistics. Lung Cancer and Personalized Medicine.

[2] Siegel RL, Miller KD, Jemal A (2017). Cancer statistics, 2017. CA Cancer J. Clin..

[3] Tsuboi M (2007). The present status of postoperative adjuvant chemotherapy for completely resected non-small cell lung cancer. Ann. Thorac. Cardiovascular Surg..

[4] Wood DE (2015). National Comprehensive Cancer Network (NCCN) clinical practice guidelines for lung cancer screening. Thorac. Surg. Clin..

[5] Klastersky J (2004). Management of fever in neutropenic patients with different risks of complications. Clin. Infect. Dis..

[6] Maeda H, Wu J, Sawa T, Matsumura Y, Hori K (2000). Tumor vascular permeability and the EPR effect in macromolecular therapeutics: a review. J. Control. Release.

[7] Zhou J, Rossi J (2017). Aptamers as targeted therapeutics: current potential and challenges. Nat. Rev. Drug Discov..

[8] Liechty WB, Peppas NA (2012). Expert opinion: responsive polymer nanoparticles in cancer therapy. Eur. J. Pharm. Biopharm..

[9] Egusquiaguirre SP, Igartua M, Hernández RM, Pedraz JL (2012). Nanoparticle delivery systems for cancer therapy: advances in clinical and preclinical research. Clin. Transl. Oncol..

[10] Tuerk C, Gold L (1990). Systematic evolution of ligands by exponential enrichment:RNA ligands to bacteriophage T4 DNA polymerase. Science.

[11] Zhao Z (2009). Recognition of subtype non-small cell lung cancer by DNA aptamers selected from living cells. Analyst.

[12] Keefe AD, Pai S, Ellington A (2010). Aptamers as therapeutics. Nat. Rev. Drug Discov..

[13] Shangguan D (2006). Aptamers evolved from live cells as effective molecular probes for cancer study. Proc. Natl Acad. Sci. USA.

[14] Zhang K (2012). A novel aptamer developed for breast cancer cell internalization. ChemMedChem.

[15] Shigdar S (2011). RNA aptamer against a cancer stem cell marker epithelial cell adhesion molecule. Cancer Sci..

[16] Ara MN (2014). An aptamer ligand based liposomal nanocarrier system that targets tumor endothelial cells. Biomaterials.

[17] Davydova AS, Vorobjeva MA, Venyaminova AG (2011). Escort aptamers: new tools for the targeted delivery of therapeutics into cells. Acta Nat..

[18] Kumari A, Yadav SK, Yadav SC (2010). Biodegradable polymeric nanoparticles based drug delivery systems. Colloids Surf. B Biointerfaces.

[19] Gaur U (2000). Biodistribution of fluoresceinated dextran using novel nanoparticles evading reticuloendothelial system. Int. J. Pharm..

[20] Lin WJ, Juang LW, Lin CC (2003). Stability and release performance of a series of pegylated copolymeric micelles. Pharm. Res..

[21] Apte RS (2008). Pegaptanib sodium for the treatment of age-related macular degeneration. Expert Opin. Pharmacother..

[22] Allen C, Maysinger D, Eisenberg A (1999). Nano-engineering block copolymer aggregates for drug delivery. Colloids Surf. B Biointerfaces.

[23] Jiang W, Kim BYS, Rutka JT, Chan WCW (2008). Nanoparticle-mediated cellular response is size-dependent. Nat. Nanotechnol..

[24] Feng S-S, Chien S (2003). Chemotherapeutic engineering: application and further development of chemical engineering principles for chemotherapy of cancer and other diseases. Chem. Eng. Sci..

[25] Shapira A, Livney YD, Broxterman HJ, Assaraf YG (2011). Nanomedicine for targeted cancer therapy: towards the overcoming of drug resistance. Drug Resist. Updat..

[26] Livney, Y. D. & Assaraf, Y. G. Rationally designed nanovehicles to overcome cancer chemoresistance. *Adv. Drug Deliv. Rev*. 65, 1716–1730 (2013). 10.1016/j.addr.2013.08.006 23954781

[27] Oh KT (2009). The reversal of drug-resistance in tumors using a drug-carrying nanoparticular system. Int. J. Mol. Sci..

[28] Livney YD, Assaraf YG (2013). Rationally designed nanovehicles to overcome cancer chemoresistance. Adv. Drug Deliv. Rev..

[29] Bar-Zeev M, Nativ L, Assaraf YG, Livney YD (2018). Re-assembled casein micelles for oral delivery of chemotherapeutic combinations to overcome multidrug resistance in gastric cancer. J. Mol. Clin. Med..

[30] Bar-Zeev Maya, Kelmansky Daniel, Assaraf Yehuda G., Livney Yoav D. (2018). β-Casein micelles for oral delivery of SN-38 and elacridar to overcome BCRP-mediated multidrug resistance in gastric cancer. European Journal of Pharmaceutics and Biopharmaceutics.

[31] Caetano-Pinto Pedro, Jansen Jitske, Assaraf Yehuda G., Masereeuw Rosalinde (2017). The importance of breast cancer resistance protein to the kidneys excretory function and chemotherapeutic resistance. Drug Resistance Updates.

[32] Fessi H, Puisieux F, Devissaguet JP, Ammoury N, Benita S (1989). Nanocapsule formation by interfacial polymer deposition following solvent displacement. Int. J. Pharm..

[33] Wang Y, Yu L, Han L, Sha X, Fang X (2007). Difunctional Pluronic copolymer micelles for paclitaxel delivery: synergistic effect of folate-mediated targeting and Pluronic-mediated overcoming multidrug resistance in tumor cell lines. Int. J. Pharm..

[34] Xin H (2010). Enhanced anti-glioblastoma efficacy by PTX-loaded PEGylated poly(varepsilon-caprolactone) nanoparticles: in vitro and in vivo evaluation. Int. J. Pharm..

[35] Li R (2009). Preparation and evaluation of PEG-PCL nanoparticles for local tetradrine delivery. Int. J. Pharm..

[36] Liu JS (2014). Enhanced brain delivery of lamotrigine with Pluronic® P123-based nanocarrier. Int. J. Nanomed..

[37] Engelberg, S., Modrejewski, J., Walter, J. G., Livney, Y. D. & Assaraf, Y. G. Cancer cell-selective, clathrin-mediated endocytosis of aptamerdecorated nanoparticles. *Oncotarget* **9**, 20993–21006 (2018). 10.18632/oncotarget.24772 PMC5940367 29765515

[38] Apodaca G (2001). Endocytic traffic in polarized epithelial cells: role of the actin and microtubule cytoskeleton. Traffic.

[39] Cabral H (2011). Accumulation of sub-100 nm polymeric micelles in poorly permeable tumours depends on size. Nat. Nanotechnol..

[40] Li R (2009). Preparation and evaluation of PEG–PCL nanoparticles for local tetradrine delivery. Int. J. Pharm..

[41] Danhier F (2009). Paclitaxel-loaded PEGylated PLGA-based nanoparticles: In vitro and in vivo evaluation. J. Control. Release.

[42] Gu. et al. Precise engineering of targeted nanoparticles by using self-assembled biointegrated block copolymers*. Proc. Natl Acad. Sci. USA*. **105**, 2586–2591 (2008). 10.1073/pnas.0711714105 PMC2268180 18272481

[43] Aravind A (2012). Aptamer-labeled PLGA nanoparticles for targeting cancer cells. Cancer Nanotechnol..

[44] Mukerjee A, Vishwanatha JK (2009). Formulation, characterization and evaluation of curcumin-loaded PLGA nanospheres for cancer therapy. Anticancer Res..

[45] Aravind A (2012). AS1411 aptamer tagged PLGA-lecithin-PEG nanoparticles for tumor cell targeting and drug delivery. Biotechnol. Bioeng..

[46] Kim SY, Shin IG, Lee YM, Cho CS, Sung YK (1998). Methoxy poly(ethylene glycol) and ϵ-caprolactone amphiphilic block copolymeric micelle containing indomethacin.: II. Micelle formation and drug release behaviours. J. Control. Release.

[47] Chen SJ (2008). Colorimetric determination of urinary adenosine using aptamer-modified gold nanoparticles. Biosens. Bioelectron..

[48] Huang, Y.-F., Chang, H.-T. & Tan, W. Cancer cell targeting using multiple aptamers conjugated on nanorods. *Anal. Chem*. **80**, 567–572 (2008). 10.1021/ac702322j 18166023

[49] Ehrlich M (2004). Endocytosis by random initiation and stabilization of clathrin-coated pits. Cell.

[50] Champion JA, Mitragotri S (2006). Role of target geometry in phagocytosis. Proc. Natl Acad. Sci. USA.

[51] Gould GW, Lippincott-Schwartz J (2009). New roles for endosomes: From vesicular carriers to multi-purpose platforms. Nat. Rev. Mol. Cell Biol..

[52] Schmidt KS (2004). Application of locked nucleic acids to improve aptamer in vivo stability and targeting function. Nucleic Acids Res..

[53] Martins AM (2009). The role of lipase and α-amylase in the degradation of starch/poly(ɛ-caprolactone) fiber meshes and the osteogenic differentiation of cultured marrow stromal cells. Tissue Eng. Part A.

[54] Dhar S, Gu FX, Langer R, Farokhzad OC, Lippard SJ (2008). Targeted delivery of cisplatin. Proc. Natl Acad. Sci. USA.

[55] Esfandyari-Manesh M (2016). Specific targeting delivery to MUC1 overexpressing tumors by albumin-chitosan nanoparticles conjugated to DNA aptamer. Int. J. Pharm..

[56] Zhang L, He Y, Ma G, Song C, Sun H (2012). Paclitaxel-loaded polymeric micelles based on poly(ɛ-caprolactone)-poly(ethylene glycol)-poly(ɛ-caprolactone) triblock copolymers: in vitro and in vivo evaluation. Nanomedicine.

[57] Shapira A, Assaraf YG, Livney YD (2010). Beta-casein nanovehicles for oral delivery of chemotherapeutic drugs. Nanomedicine.

[58] Xin H (2010). Enhanced anti-glioblastoma efficacy by PTX-loaded PEGylated poly(ε-caprolactone) nanoparticles: in vitro and in vivo evaluation. Int. J. Pharm..

[59] Bar-Zeev, M., Assaraf, Y. G. & Livney, Y. D. β-casein nanovehicles for oral delivery of chemotherapeutic Drug combinations overcoming P-glycoprotein-mediated multidrug resistance in human gastric cancer cells. *Oncotarget* **7**, 23322–23334 (2016). 10.18632/oncotarget.8019 PMC5029629 26989076

[60] Bellare JR, Davis HT, Scriven LE, Talmon Y (1988). Controlled environment vitrification system: an improved sample preparation technique. J. Electron Microsc. Tech..

[61] Edelman, R., Assaraf, Y. G., Levitzky, I., Shahar, T. & Livney, Y. D. Hyaluronic acid-serum albumin conjugate-based nanoparticles for targeted cancer therapy. *Oncotarget* **8**, 24337–24353 (2017). 10.18632/oncotarget.15363 PMC5421851 28212584

